# On Dilatation by Fluid Pressure in Stricture of the Urethra

**Published:** 1841-10

**Authors:** James Arnott


*On Dilatation by Fluid Pressure in Stricture of the Urethra.* By JAMES ARNOTT, M. D.—Although our knowledge of the pathology of stricture of the urethra has been much extended by the labors of Hunter and others, the treatment of this very common and distressing disease differs at the present day in no very material circumstance from that which was followed two hundred years ago. In the works of Wiseman, published in the reign of Charles the II., the various practices now had recourse to will be found described. He mentions the use both of metallic and soft bougies; the application of caustic is noticed, a practice which was revived by Hunter; and cases are related in which the operation of opening the urethra behind the stricture was performed, instead of puncturing the bladder—an expedient of which the late eminent Sir Astley Cooper has been deemed the original proposer.

Unfortunately, this stationary condition, during the progress of almost every other department of surgery, has not preceeded from the treatment of stricture having attained perfection. On the contrary, it is acknowledged to be an opprobrium of the art. The means employed are admitted, by conscientious and intelligent surgeons, to be, in almost every case, but palliative; and although stricture may generally be much relieved by such means applied from time to time, it cannot be denied that the irritation which accompanies organic changes in a part of so much sensibility as the urethra, will, by long continuance, often produce other disease in the neighboring organs of the urinary and generative systems, which is sure to embitter, if it does not shorten, the life of the sufferer.

It is now many years since I introduced to the profession an account of practices in the treatment of stricture which I had had sufficient experience to recommend as substitutes for the very imperfect and sometimes hazardous measures in common use. But because the apparatus recommended was of rather a complicated description, as compared with that usually employed, and because part of it was constructed on mechanical principles, with which surgeons generally were not familiar, it has either not been used at all in this country, (where the French modifications of plans of treatment I had proposed in impervious stricture, and of a new method of applying caustic, are almost unknown,) or in so imperfect and erroneous a manner, as to disappoint expectation.

The purpose of this paper is to describe a modification, which I have lately contrived, of the instrument employed in the dilatation of stricture, combining the essential requisites, for general use, of simplicity of construction and easiness of application; and I cannot doubt, from its great and manifest superiority over the means commonly had recourse to, in the degree of relief afforded by it, and the safety and quickness with which this is obtained, that it only requires to be known to be immediately adopted.

Dilatation of stricture has been effected in two ways: by instruments which operate on the principle of the wedge, opening the constricted part as they advance in the canal, of which description are bougies and sounds; and by instruments which are themselves capable of distension, and which, by being made to enlarge in diameter whilst within the stricture, exert their dilating force from the centre directly outwards, or, as it may be termed eccentrically. Amongst the principal advantages of eccentric dilatation over that of the wedge, when effected by a proper apparatus, are—that instruments so operating, having no tendency to stretch or tear the urethra in front of the stricture, by pushing on the stricture after having passed partially through it, the surgeon is enabled by their means to use greater force (if required) with safety, than with rigid bougies and sounds, which have this tendency; that there is no danger, from the opposition to the passage of the instrument being erroneously attributed to the stricture instead of the wrong direction of its point, of the surgeon's piercing the side of the canal, and causing effusion of urine, or false passage; that the dilatation being effected without irritation from friction, and following the yielding structure, may be rapidly made; that the whole of a long stricture may be dilated at once, or several strictures are acted upon at the same time, instead of the action being nearly confined, as in the case of the bougie, to the front or face of the first stricture; and that, from the power of enlarging the instrument in the interior of the canal to any size, the dilatation of the diseased part may be carried to any greater extent than the diameter of the outer orifice of the urethra, so as to afford the best means of effecting a permanent cure.

The apparatus used for dilatation, on the principle just explained, consists essentially of a strong membranous tube of fixed dimensions, which is placed, in its empty or collapsed state, within the stricture, and then injected with fluid. I have used such a fluid dilator, with various modifications, according to peculiarities of cases. The form of instrument most easily constructed and applied, and which has not as yet been described, is merely a varnished silk tube of the required diameter, and of a length to extend from the orifice to a little beyond the stricture, closed at one end, and having a small metalic connecting piece at the other, into which the injecting syringe may be screwed. This tube, by means of a slight coating of waxy composition, is, for the purpose of passing easily, rolled into the form of a common plaster bougie; and when it is not required to be of very small diameter in its collapsed state, the requisite stiffness may be given to it by rolling it upon a small catgut or stilet. A woven silk tube properly varnished would be perfectly water-tight; but this is of less importance, as a thick mucilaginous liquid will not escape but very slowly from a very imperfect tube made by sewing together the edges of a riband. This instrument, which may be described as a dilatable bougie, is as durable, and may be made at as little expense, as any instrument used in the treatment of stricture.

In keeping up distension, by means of a dilator rendered impervious to fluid by gut or caoutchouc, instead of the stopcock recommended in former instructions on the subject, a contrivance may be employed for fixing the pistion of the syringe, when the required degree of pressure has been made, as by a cord passing through the ring at the end of the piston rod, or by a screw. When the piston is depressed by a screw (which may constitute the piston rod) the patient can himself increase or moderate the pressure with the greatest facility. If the distensible tube be made of strong silk, it may be thus gradually distended until it becomes as hard as a cylinder of wood. A connecting flexible tube of silk and caoutchouc between the metallic part of the dilator and syringe, prevents any jerking motion of the instrument in the act of screwing on the syringe, and is a convenient index of the degree of pressure applied.

In other applications of the fluid dilator, as in the cure of stricture of the rectum, and in the operation of slowly dilating the male or female urethra for the extraction of calculi, a long connecting tube of this description, bringing the screw which regulates the pressure conveniently to the hand of the patient, would render the apparatus very complete. I have shown in the appendix to the late edition of my work on Stricture and Stone, that the advantage of *slow* dilatation of the male urethra must have frequently occurred to the operators by the Marian method, who professed to follow nature in their proceedings, as it has occurred independently to several surgeons of late years; but that the want of any instrument which could fulfil the indication must have prevented the success of any attempt of the kind. The equable, elastic, and controllable nature of fluid pressure, makes a dilator, judiciously constructed on this principle, incomparably superior to any other means that has been employed for the purpose; and furnishes us with a method of extracting urinary calculi, which, if I am not much deceived, will soon supercede the present painful and dangerous operations.

When stricture is to be dilated beyond the diameter of the orifice of the urethra, it is necessary to modify the instrument which has been mentioned above. The distension may be confined to the diseased part by placing a wide silk tube within another shorter tube of smaller diameter, or by passing it through a wide silver or elastic tube previously inserted as far as the stricture. In cases of very narrow stricture, only admitting instruments of the smallest size, a dilator may be passed through such a conducting tube, consisting either of a single or double piece of narrow gut dried in a compressed form, or of a silk tube rolled upon itself, and rendered sufficiently rigid by means of thick mucilage. It is unnecessary in these cases to have a distensible tube of the whole length of the conductor; a small bit tied upon the end of a long flexible tin tube, connecting it with the syringe, is sufficient.

In mentioning this mode of conveying a small instrument to narrow strictures by means of a conducting tube, I am led to notice a controversy which has been continued through several late numbers of the Medical Gazette, respecting the invention of what has been termed "the compound catheter." Dr. Buchanan, who claims the originality of this proposal, does not appear to be conversant with the modern French writers on urinary diseases, or he would have found that the plan of conducting small instruments through others of larger size is noticed in most of the works on that subject which have appeared in France during the last twenty years. But it is contended that Dr. Buchanan's instrument is more than a mere modification of former suggestions; that the principle of it extends further: the smaller instrument, it is said, is not only conducted, but a way is prepared for it through the stricture by a dilatation effected by the pressure of the ends of the outer canulæ. Had a reference been made to the work from which M. Ducamp borrowed so liberally, instead of the treatise of M. Gerdy, who in this matter professes merely to follow Ducamp, it would have been discovered that there is no greater novelty in this idea of previous dilatation, than in that of conducting. In my Treatise on Stricture of the Urethra, (p. 133, 2d edition,) the following is mentioned amongst other means to be resorted to in cases of difficulty;—"The plan which I have recommended above, of passing a large canula (which in this case has a rounded end) enclosing an instrument down to the stricture, is very applicable here; pushing the canula against the stricture opens it, while the small bougie or catheter within is ready to be passed through."—*Lon. Med. Gaz.*

